# Institutional Impact of EVAR's Incorporation in the Treatment of Abdominal Aortic Aneurysm: a 12 Years' Experience Analysis

**DOI:** 10.5935/1678-9741.20160028

**Published:** 2016

**Authors:** Rui Machado, Inês Lopes Antunes, Pedro Oliveira, Carlos Pereira, Rui de Almeida

**Affiliations:** 1 Hospital de Santo António - Centro Hospitalar do Porto, Porto, Portugal; 2 Instituto de Ciências Biomédicas Abel Salazar (ICBAS), Porto, Portugal

**Keywords:** Aortic Aneurysm, Abdominal, Endovascular Procedures, Vascular Surgical Procedures, Health Risk

## Abstract

**Introduction:**

Endovascular aneurysm repair (EVAR) was introduced as a less aggressive treatment of abdominal aortic aneurysms (AAA) for patients ineligible for open repair (OR).

**Objective:**

To analyze EVAR's incorporation impact in the treatment of infra-renal abdominal aortic aneurysms in our institution.

**Methods:**

A retrospective study of the patients with diagnostic of infra-renal AAA treated between December 2001 and December 2013 was performed. The choice between EVAR and OR was based on surgeon's experience, considering patient clinical risk and aneurysm's anatomical features. Patients treated by EVAR and by OR were analyzed. In each group, patient's and aneurysm's characteristics, surgical and anesthesia times, cost, transfusion rate, intraoperative complications, hospital stay, mortality and re-intervention rates and survival curves were evaluated.

**Results:**

The mean age, all forms of heart disease and chronic renal failure were more common in EVAR group. Blood transfusion, surgical and anesthesia times and mean hospital stay were higher for OR. Intraoperative complications rate was higher for endovascular aneurysm repair, overall during hospitalization complication rate was higher for open repair. The average cost in endovascular aneurysm repair was 1448.3€ higher. Re-interventions rates within 30 days and late re-intervention were 4.1% and 11.7% for endovascular aneurysm repair *versus* 13.7% and 10.6% for open repair.

**Conclusions:**

Two different groups were treated by two different techniques. The individualized treatment choice allows to achieve a mortality of 2.7%. Age ≥80 years influences survival curve in OR group and ASA ≥IV in EVAR group. We believe EVAR's incorporation improved the results of OR itself. Patients with more comorbidities were treated by endovascular aneurysm repair, decreasing those excluded from treatment. Late reinterventions were similar for both techniques.

**Table:** 

Abbreviations, acronyms & symbols	
AAA	= Abdominal aortic aneurysm
COPD	= Chronic obstructive pulmonary disease
CT	= Computerized tomography
EVAR	= Endovascular aneurysm repair
OR	= Open repair

## INTRODUCTION

Abdominal aortic aneurysms (AAA) is a relatively common disease. Its prevalence increases with age. The main risk factors are age older than 65 years, male gender and smoking history^[1]^. As the aneurysm size increases, there is the risk of rupture^[2]^. Although some patients may present vague symptoms such as abdominal or back pain, the majority of aneurysms remain asymptomatic until rupture^[3]^, which has a mortality rate about 85%^[4]^. The goal of treatment is to exclude the aneurysm before rupture occurs^[5]^.

Treatment by open repair (OR) is practiced since 1951. In the 90s, endovascular aneurysm repair (EVAR) was introduced as a less invasive method^[6]^, originally developed for patients considered ineligible for OR^[7]^. Its introduction aimed to improve the care provided to the patient offering a therapeutic possibility with less surgical aggression initially without thinking that could compete with OR. Older patients with major comorbidities previously excluded from treatment have become candidates for EVAR allowing a reduction in the number of patients without conditions for treatment over the years. But the selection of the treatment method to each patient is not always clear and is based on the results of randomized studies, national/international series and individual choice based on surgeon's opinion, considering patient clinical risk and aneurysm features.

There is strong evidence of OR's durability, but there are few long-term results for EVAR. So, there is still an uncertainty concerning EVAR's durability and its overall long-term efficacy when compared to OR^[8]^. This lack of knowledge about the future behavior implies a greater need for clinical and imaging surveillance which could represent higher costs.

Several studies compared EVAR to OR particularly regarding the perioperative and long-term mortality, re-intervention rates and cost-effectiveness, sometimes with diverging results.

The Clinical Practice Guidelines of the European Society of Vascular Surgery suggest that vascular surgical referral centers must have an operative mortality for elective OR less than 5% and for EVAR less than 2%^[9]^.

Having regard to selection of the best treatment to be used for each patient and knowing that these treatment methods are complementary and not competitive, the purpose of this study is to analyze the impact of EVAR's incorporation in the treatment of infra-renal AAA in our institution.

## METHODS

A retrospective study of the patients with the diagnostic of infra-renal AAA treated in our institution between December 2001 and December 2013 was performed. Patients with the diagnosis of infra-renal AAA with diameter equal or superior to 5cm and patients with infra-renal AAA with diameter inferior to 5cm but with iliac aneurysms with diameter equal or superior to 3cm were included in our study.

The choice between EVAR and OR was individualized for each patient, based on surgeons' opinion, considering patient clinical risk and aneurysm's anatomical features.

During this period, a total of 292 patients were treated in our institution with the diagnostic of infra-renal AAA, 171 (58.6%) by EVAR and 121 (41.4%) by OR.

We analyzed the group of patients treated by EVAR and by OR and, for each group, we studied patient's and aneurysm's characteristics, surgical and anesthesia average times, need for blood transfusion, intraoperative complications, mean hospital stay, re-intervention rates (within 30 days and after), mortality rate (during hospitalization and within 30 days) and survival curves. We studied costs associated to EVAR and to OR and the relation between costs and age and costs and American Society of Anesthesiologists (ASA) classification.

The mean follow-up time was 32.4 months. The follow-up was performed with computerized tomography (CT) in the 3^rd^ and 9^th^ month after treatment and then yearly or every time it seems clinically relevant.

The statistical method used evaluates the normal distribution of the continuous variables using the Kolmogorov-Smirnov test. The comparison between two groups of patients was based on Student's *t* test for variables that approximately followed a normal distribution and the Mann-Whitney-Wilcoxon test in the event that the assumptions of normality or equality of variances were not observed. Comparison of more than two groups was based on analysis of variance and the Kruskal-Wallis test, when the assumptions of normality and homogeneity of variances were absent.

## RESULTS

**Fig.1:**
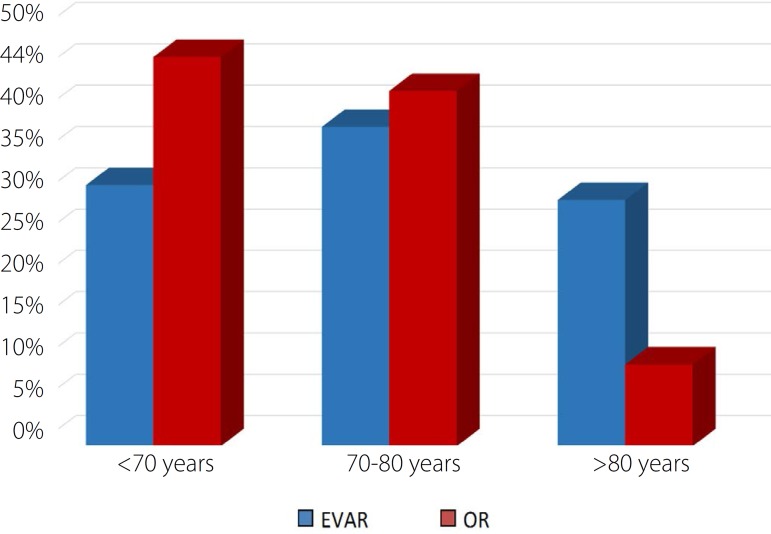
Distribution of the type of treatment according to age group.

The mean age was 74.1±8.9 years in the EVAR group and 69.6±8.7 years in the OR group, this variable proved to have statistical relevance (*P*<0.001). The treatment performed according to the age group was also studied (Figure 1). In this sense, the patients were divided into three groups: with age up to 70 years, between 70 and 80 years and above 80 years. For each group, respectively, the treatment was EVAR in 31.6%, 38.6%, 29.8% and OR in 47.1%, 43%, 9.9%. A statistically significant relationship was observed (*P*<0.001) with the younger group most often treated by OR and the older one most often by EVAR.

In relation to gender, 94.2% of patients in the EVAR group were male and 5.8% were female. In OR group, 95% were male and 5% were female. There was no relationship with statistical significance among variables (*P*=0.478).

Regarding the presence of aortic atherosclerotic disease risk factors we studied hypertension, diabetes *mellitus*, dyslipidemia, cerebrovascular disease, peripheral arterial disease and active/non-active smoking. No statistically significant relationship was observed for high blood pressure (84.2% in the EVAR group *vs.* 87% in the OR group, *P*=0.610), non-active smoking (58.8% in EVAR *vs.* 57% in OR; *P*=0.807), dyslipidemia (67.6% in EVAR *vs.* 61.4% in OR, *P*=0.134), diabetes mellitus (18.2% in EVAR *vs.* 11.4% in OR, *P*=0.134) and peripheral arterial disease (18.2% in EVAR *vs.* 19.3% in OR, *P*=0.877). A statistically significant relationship was observed for active smoking (16.5% in EVAR *vs.* 31.5% in OR, *P*=0.004) and for cerebrovascular disease (19.4% in EVAR *vs.* 10.5% in OR, *P*=0.048).

Associated diseases (heart, lung or kidney diseases) were studied for each group. Regarding the presence of cardiac disease studied were higher in the EVAR group with a statistically significant relationship with ischemic cardiac disease present in 53% of the patients in the EVAR group *vs.* 40.4% of those in OR group (*P*=0.039), valvular disease present in 27% of those submitted to EVAR *vs.* 4.4% of those in the OR group (*P*<0.001), dysrhythmia present in 37.2% in the EVAR *vs.* 12.4% in the OR group (*P*<0.001) and cardiac insufficiency present in 45.1% of the patients in the EVAR group *vs.* 20.2% in the OR group (*P*<0.001). Regarding pulmonary disease, chronic obstructive pulmonary disease (COPD) was present in 24.1% of patients in the EVAR group and in 26.8% in the OR group, no statistically significant relationship was observed (*P*=0.672) and respiratory failure was present in 5.6% of the patients in the EVAR group *vs.* 0.9% in the OR group, an almost statistically significant relationship was observed (*P*=0.051). Regarding renal disease, a statistically significant relationship was observed for the presence of chronic renal insufficiency (21.3% of patients treated by EVAR *vs.* 8.8% in OR, *P*=0.007) but wasn't observed for chronic renal insufficiency in hemodialysis replacement therapy (1.8% of the patients in the EVAR *vs.* 0 in the OR group, *P*=0.274) or chronic renal insufficiency in kidney transplantation replacement therapy (2.5% of those in the EVAR *vs.* 0.9% in the OR group, *P*=0.651).

As regards the ASA physical status classification our patients were in one of three categories: ASA II (mild systemic disease), ASA III (severe systemic disease) or ASA IV (systemic disease threatening life). The frequency was, respectively, 15.6%, 71.3% and 13.1% in the EVAR group and 30.3%, 62.4% and 7.3% in the OR group. A statistically significant relationship was observed (*P*=0.001) with patients classified as class II more commonly treated by OR and class IV by EVAR.

Aneurysm characteristics were also studied. Regarding the anatomical type divided into aortic, bilateral aorto-iliac, right aorto-iliac and left aorto-iliac this was respectively 65.5%, 11.7%, 15.2% e 7.6% in the EVAR group and 80.2%, 9.0%, 8.1% e 2.7% in the OR group. A statistically significant relationship was observed (*P*=0.045) with aortic aneurysms treated most commonly by OR and aorto-iliac aneurysms (right and left) by EVAR.

Regarding the aneurysm etiology, divided into degenerative, inflammatory and other etiology, was respectively 93.5%, 5.3% and 4.1% in the EVAR group and 97.3%, 2.7% and 0 in the OR group, no statistically significant relationship was observed (*P*=0.092). The aneurysm etiology was also studied dividing into only two etiologies degenerative and inflammatory, which represent respectively 97.5% and 2.5% in the EVAR group *vs*. 97.3% and 2.7% in the OR group, no statistically significant relationship was observed (*P*=0.001).

Aneurysm morphology, divided into fusiform and saccular was respectively 94.7% and 5.3% in the EVAR group *vs.* 72.3% and 27.7% in the OR group, a statistically significant relationship was observed (*P*<0.001) with saccular aneurysms most commonly treated by OR.

As regards the aneurysm diameter, it was 62.4±14.8 mm in the group treated by EVAR and 64.8±15.7 mm in the OR group. No statistically significant relationship (*P*=0.201) was observe.

Blood transfusion was needed in 23.1% in the EVAR group *vs.* 77.6% in the OR group, a statistically significant relationship was observed between these variables (*P*<0.001).

The mean anesthesia time was 174.4±63.2 minutes in the EVAR group and 292.6±79.5 minutes in the OR group, a statistically significant relationship was observed between these variables (*P*<0.001). The mean surgical time was 102.7±48.4 minutes in the EVAR group and 190.1±61.5 minutes in the OR group, a statistically significant relationship was observed between these variables (*P*<0.001).

Regarding EVAR's intraoperatory complications, we considered endoleaks requiring additional treatment, arterial dissection/thromboses or other situations that require some additional intervention. In OR group, we considered vascular or visceral damage. Intraoperative complications rate was 23.4% in the EVAR group and 14.4% in the OR group, a statistically significant relationship was observed (*P*<0.001). The overall rate of complications during hospitalization was higher in the OR group (38% in the OR group *vs.* 10.8% for EVAR), with a statistically significant relationship (*P*<0.001).

We analyzed the costs associated with an EVAR and OR procedures (Table 1). The average cost in EVAR was 1.448,3€ higher in comparison to OR. When we studied the relation between costs and age (Table 2) and costs and ASA classification (Table 3), no statistically significant relation was observed regarding age, but for both groups a statistically significant relation was observed between costs and ASA classification with patients classified ASA IV or above implying a significant higher cost in EVAR and in OR.

**Table 1:** Global costs.

	Mean	Median	Std. Deviation	Minimum	Maximum	Percentil 25	Percentil 75
EVAR	11,404.00 €	10,387.70 €	4,489.40 €	9,081.50 €	50,779.70 €	9,979.30 €	11,141.70 €
Open repair	9,955.70 €	7,189.00 €	10,062.90 €	3,819.00 €	95,144.00 €	5,635.00 €	10,561.70 €

**Table 2:** Costs and age.

		Mean	Median	Std. Deviation	Minimum	Maximum	Percentil 25	Percentil 75	ES
EVAR	<70 years	11,658 €	10,226.80 €	6,157.80 €	9,270 €	50,779.70 €	9,867.50 €	10,996.70 €	N
	70-80 years	11,110.30 €	10,484.70 €	2,163 €	9,433 €	20,979 €	10,017.80 €	11,317 €	
	>80 years	11,521.80 €	10,371.30 €	4,752.80 €	9,081.50 €	40,240.70 €	10,007.80 €	11,068.40 €	
Open repair	<70 years	9,478.80 €	6,218.90 €	12,925.10 €	3,839.90 €	95,144 €	5,189.50 €	8,217.30 €	N
	70-80 years	10,836.60 €	9,370.80 €	6,549.80 €	3,819 €	36,003 €	6,180.40 €	13,155.80 €	
	>80 years	8,607.60 €	6,534.80 €	3,911.50 €	4,592 €	14,340 €	5,476.60 €	12,424.40 €	

**Table 3:** Costs and ASA classification.

		Mean	Median	Std. Deviation	Minimum	Maximum	Percentil 25	Percentil 75	ES
EVAR	ASA II	11,356.50 €	9,990 €	6,185.30 €	9,270 €	40,240.70 €	9,694.20 €	10,431.80 €	Y
	ASA III	10,776.70 €	10,371.30 €	1,849 €	9,081.50 €	22,643.20 €	10,012 €	10,870.80 €	
	ASA IV	14,725.20 €	11,926.20 €	8,858.40 €	9,644.30 €	50,779.30 €	10,631 €	15,539.30 €	
Open repair	ASA II	9,613.50 €	6,471.10 €	7,434.20 €	4,174.20 €	36,003 €	5,707.30 €	10,367.80 €	Y
	ASA III	8,758 €	7,783.20 €	4,469.80 €	3,819 €	24,901.70 €	5,557.30 €	10,829.20 €	
	ASA IV	21,729.80 €	9,762.20 €	30,464.90 €	4,761.50 €	95,144 €	6,653.10 €	23,607.20 €	

The mean duration of hospitalization was 6.3±7.1 days for the EVAR group and 12.9±16.6 days for the OR group, a statistically significant relationship was observed between these variables (*P*<0.001).

**Fig. 2:**
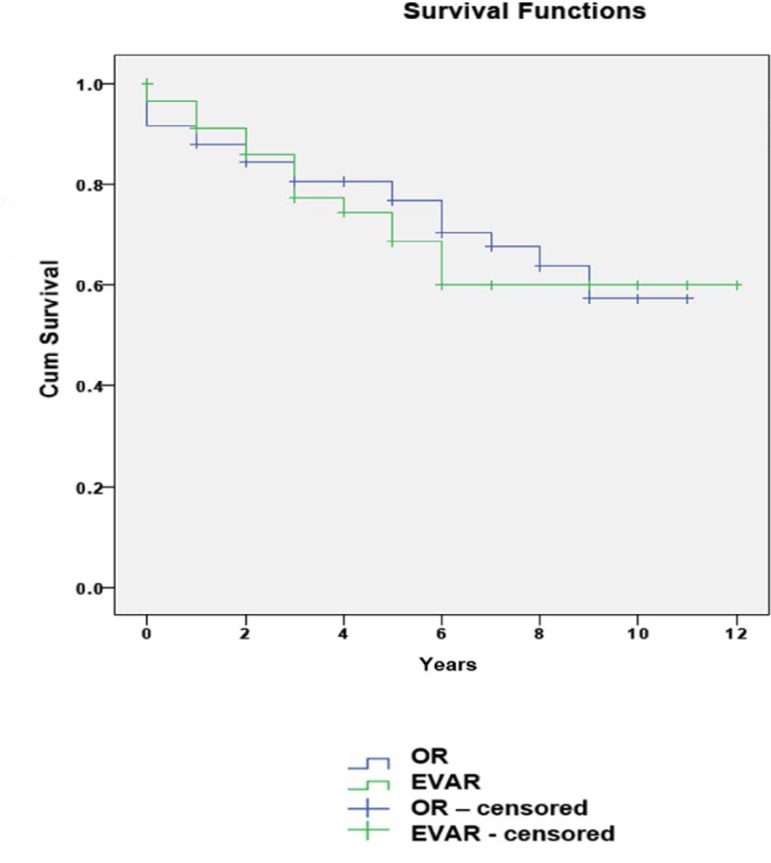
Survival curves in EVAR and OR groups.

**Fig. 3:**
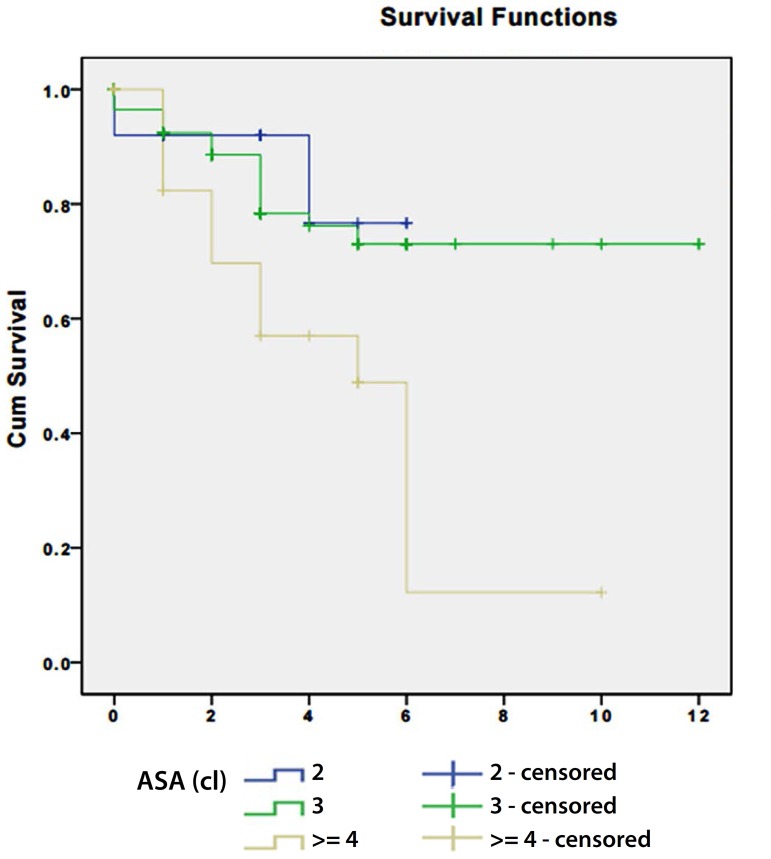
Survival curves in EVAR group according to ASA classification.

**Fig. 4:**
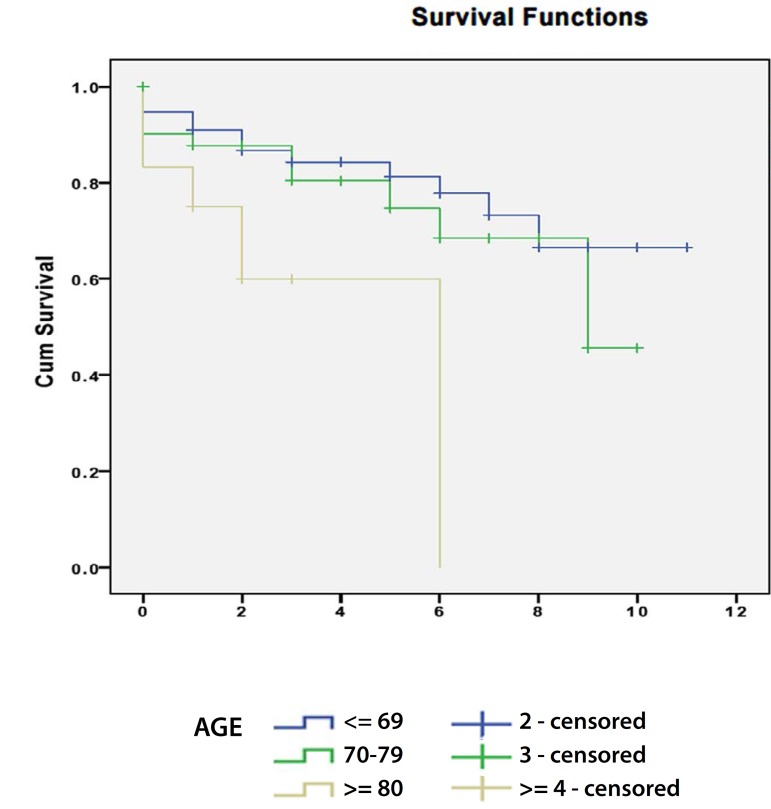
Survival curves in OR group according to age group.

The overall mortality during hospitalization with the use of both techniques was 2.7% (1.2% in the EVAR group and 5% in the OR group), no statistically significant relationship was observed (*P*=0.07). Mortality within 30 days was 1.2% in the EVAR group and 5% in the OR group. We also studied the survival curves for EVAR and for OR (Figure 2). The median survival was 8.5 years with a standard deviation of 0.5 (95% CI - 7.6 to 9.5) in the EVAR group and 8.2 years with a standard deviation of 0.4 (95% CI- 7.3 to 9.0) in the OR group. We analyzed relation between survival curves and ASA classification and age. We found that in EVAR group the median survival in the group classified as ASA II was 5.2 years with a standard deviation of 0.4 (95% CI - 4.4 to 6), in the group classified as ASA III was 9.4 years with 0.5 standard deviation (95% CI - 8.4 to 10.5) and in the group classified as ASA IV or higher was 4.6 years with a standard deviation of 0.8 (95% CI - 3.2 to 6.1), a statistically significant relation was observed with patients classified as ASA IV or more having a lower mean survival (Figure 3), no statistically significant relation was observed in the OR group. When we analyzed the relation between age and survival curves we realized that, in the OR group (Figure 4) the median survival for patients under 70 years was 8.7 years with a standard deviation of 0.6 (95% CI - 7.6 to 8.9), in the group between 70 and 80 years was 7.5 years with a standard deviation of 0.6 (95% CI - 6.3 to 8.7) and in the group of patients older than 80 years was 4 years with a standard deviation of 0.9 (95% CI - 2.2 to 5.8), statistically significant relationship was observed patients older than 80 years has a lower mean survival in comparison to younger groups, in EVAR group no statistically significant relationship was observed.

Regarding re-intervention rate within 30 days was 4.1% for EVAR and 13.7% for patients treated by OR. Concerning EVAR, 28.6% of complications were related to type 1 endoleak, 14.3% thrombosis with need for an axillary-femoral bypass and 57.1% wound complications. With regard to the OR, of all the re-interventions within 30 days, 24.7% represented drain of retroperitoneal haematoma, 18.9% exploratory laparotomy, 18.9% revascularization surgery, 6.6% colectomy, 13% wound surgery and 18.9% incisional hernia repair. With a mean followup of 32.4 months our protocol follow-up involves CT in the 3^rd^ and 9^th^ month after treatment and then yearly besides, CT was also performed every time it seems clinically pertinent. After 30 days, re-intervention rate was 11.7% for EVAR and 10.6% for OR, no statistically significant relation was observed between these variables (*P*=0.56). Concerning EVAR, of all the re-interventions after 30 days, 72.6% were related to type 1 endoleak, 4.8% to type 2 endoleak, 9.5% to type 3 endoleak and 9.5% because of endograft branch thrombosis. In the OR group, 90.9% were for incisional hernia repair and 9.1% (1 case) correction of a false aneurysm.

## DISCUSSION

From an overall assessment of this population, we conclude that the characteristics of patients treated by EVAR and by OR are different. Patients treated by EVAR are generally older and with more associated diseases. To point out that when we studied chronic renal insufficiency in replacement therapy we haven't observed any relation probably due to the small number of patients treated. In fact only 3 patients on hemodialysis were treated and all of them were treated by EVAR. Only 5 renal transplant patients were treated, 4 by EVAR and 1 by OR. These observations are in agreement with our results when we studied ASA classification. "The Dutch Randomised Endovascular Aneurysm Management Trial", 2005, (DREAM trial) and the "*Anevrysme de l'aorte abdominale, Chirurgie versus Endoprothese*" (ACE trial) are two randomized studies that also studied ASA classification in patients included (Table 4). In these studies, the majority of patients selected for treatment belonged to class II of ASA classification (mild systemic disease). In contrast, the majority of patients treated in our institution belong to class III (severe systemic disease). Additionally, in DREAM trial there were no patients classified as class IV and they represent only 1.3% of the patients treated, all of them by EVAR, in ACE trial. It could be explained with the fact that in DREAM trial only patients eligible for both treatments were included, this can eventually have conditioned that some patients classified as ASA IV were considered not eligible for OR and so not included in the trial. The ACE trial only included relatively good-risk patients and all patients classified as ASA IV were treated by EVAR. These results reinforce EVAR as an option for patients with ASA classification III or IV whom can now be treated with less risk^[10]^.

**Table 4:** ASA classification of the patients treated.

		ASA I - Healthy Patient (%)	ASA II - Mild Systemic Disease (%)	ASA III - Severe Systemic Disease (%)	ASA IV - Systemic Disease Threatening Life (%)
Our Institution	EVAR	-	15.6	71.3	13.1
	OR	-	30.3	62.4	7.3
DREAM trial	EVAR	21.6	69.6	8.2	-
	OR	25.3	60.9	13.8	-
ACE trial	EVAR	10.7	66.0	22.7	1.3
	OR	8.0	59.7	32.2	-

Regarding to gender, our results demonstrate the equal offer of both techniques to both genders and point out the higher prevalence of the disease in male gender as described in the literature.

When we studied the aneurysm's etiology (divided into degenerative, inflammatory and others) no statistically significant relationship was observed but it should be noted that all aneurysms classified as other etiologies were treated by EVAR. As for aneurysm size measured by its diameter no differences were observed in both groups proving that the therapeutic indication is independent of the technique used.

Regarding blood transfusion necessity we observed a clear advantage in the EVAR. We observed almost a reversal in necessities with 76.4% in the EVAR group not requiring transfusion *vs.* 77.6% in the OR group requiring blood transfusion. At a time when the availability of blood is scarce this is of great importance. It also opens a door for those who for religious reasons do not accept transfusion of blood products.

As regards time consumption in the operating room and surgeon's time consumption measured respectively by the duration of anesthesia and the surgery time there was a clear advantage in the treatment by EVAR with reduction of both times. Relatively to the overall hospital stay there was also a clear advantage in the EVAR group (6.6 days less than for OR). At a time when reducing inpatient bed is expected, EVAR can be useful. The average cost in EVAR was 1.448,3€ higher in comparison to OR. ASA classification equal or higher than 4 was associated with higher costs in both groups.

Prospective randomized trials as "The United Kingdom Endovascular Aneurysm Repair Trial 1" (EVAR 1 trial), "Open *versus* Endovascular Repair Veterans Affair Cooperative Study" (OVER trial) and the already cited ACE and DREAM trials have also recognized a reduced in the need for transfusion, shorter surgical time and shorter duration of hospitalization among patients treated by EVAR when compared to those treated by open repair^[11-13]^.

**Fig. 5:**
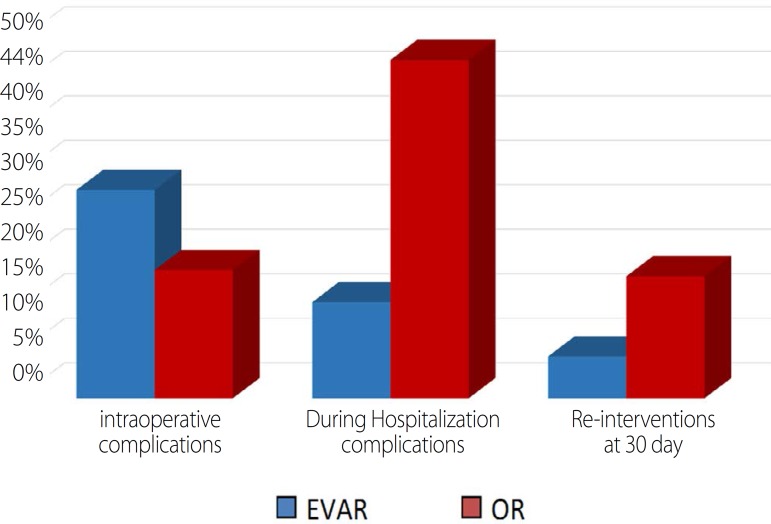
Complications and re-interventions according to the type of treatment.

Regarding complications (intraoperative and during hospitalization) only the first shows a tendency to be more numerous in the EVAR group (Figure 5). The overall rate of complications during hospitalization was significantly higher in the OR group and we concluded that most of these complications were medical and whose treatment was also medical. Actually OR showed a rate of complications during hospitalization of 38%, but the rate of re-interventions within 30 days was only 13.7% which points out that most of the complications did not require re-intervention. In comparison, the EVAR had a lower rate of complications during hospitalization (10.8%) and a lower rate of re-interventions within 30 days (4.1%).

Regarding mortality, the use of both techniques allowed to reach a global mortality during hospitalization of 2.7% (1.2% in the EVAR group and 5% in the OR group). Although not statistically significant, the mortality difference of the two groups tends to be significant (*P*=0.07). Considering that the EVAR group has a greater clinical risk as we have already concluded, we can infer benefit in terms of early mortality that this technique arrived. The Table 5 shows the mortality rates obtained in our institution and those published in some prospective randomized studies (EVAR 1, DREAM, OVER and ACE trials). Our mortality rates are similar of those obtained in those prospective randomized trials. In 2011, Mani et al.^[14]^ have published "Treatment of Abdominal Aortic Aneurysm in Nine Countries 2005-2009: A Vascunet Report", where the biggest international registration of patients with the diagnosis of AAA treated was analyzed. This registration involves nine countries record (seven nationally - Denmark, Hungary, Italy, Norway, Sweden, Switzerland and the United Kingdom and two regional - Australia and Finland). Part of the results is shown in Table 6 where we made a comparison with the results obtained in our institution. We can conclude that our findings are similar to those published in this international series.

**Table 5:** Intraoperative/during hospitalization mortality rate.

	Patients treated by EVAR (%)	Patients treated by Open Repair (%)
Our Institution	1.2	5
EVAR 1	1.7	4.7
DREAM	1.2	4.6
OVER	0.5	3.0
ACE	1.3	0.6

**Table 6:** Results obtained from Mani et al.^[14]^ regarding treatment of abdominal aortic aneurysm in nine countries 2005 e 2009: A Vascunet Report 2011 and in our institution.

	National							Regional		
Denmark	Hungary	Italy	Norway	Sweden	Switzerland	United Kingdom	Australia	Finland	Our Institution
A	2500	269	9107	2707	4134	1814	8789	1814	293	292
B	2005-2009	2008-2009	2007-2009	2005-2008	2005-2009	2005-2008	2005-2009	2005-2009	2007-2009	2001-2013
C	71.1	68.3	72.6	72.2	72.1	70.8	73.6	74.65	71.1	74.1 1
D	23.8	17.5	49	29	43.9	37.4	49.4	56	14.7	58.6
E	NR	6.2	NR	6.5	6.4	NR	7.1	6.5	6.4	6.24 1
F	1.2	4.3	0.9	0.3	1.9	2.6	1.8	1.3	2.3	1.2
G	4	2.3	2.2	2.7	3.2	3.6	5.3	3.8	4.4	5

A=number of cases; B=years of study; C=mean age (years); D=EVAR rate (%); E=mean aneurysm diameter (cm), F=operatory mortality rate for EVAR (%); G=operatory mortality rate for open repair (%);

(1) =for the group of patients treated by EVAR; NR=not reported

Long-term mortality rate associated with the two techniques has been widely studied. The EVAR 1, DREAM and OVER trials were in agreement in getting an early benefit in the perioperative mortality with EVAR but this benefit is lost during follow-up and no differences exist between the two groups in the long-term treatment. In the EVAR 1 trial the authors consider that the loss of this initial benefit throughout the study is at least in part, due to late endograft ruptures. The authors also believe that EVAR is associated with a higher rate of graft complications, more re-interventions and higher cost. A similar conclusion was presented in the DREAM and ACE trials. In contrast, the OVER trial revealed no significant difference in re-intervention rates in the both groups. In our institution the re-intervention rate after 30 days was not significantly different between the two groups corroborating the OVER trial and going against EVAR 1, DREAM and ACE trials.

## CONCLUSION

The selection of the treatment method to be used is not clear. At one extreme, we have a young patient with low clinical and anatomic risk and a complex aneurysm anatomy for which OR is the election. At the other extreme, we have an elderly patient with high clinical/anatomical risk with good aneurysm anatomy and EVAR is the election. But in most situations in clinical practice these characteristics are mixed and hinder the decision.

Our study showed that the two groups have different clinical conditions that make a comparison difficult. The EVAR group is a presents major clinical risk as demonstrated and it can lead to increased mortality during follow-up of these patients not necessarily related to its AAA.

Assessing the institutional impact of the EVAR's introduction in the treatment of patients with infra-renal AAA, we conclude that this method allowed the achievement of an overall during hospitalization mortality of 2.7% (1.2% for EVAR and 5% for OR), allowing us to achieve the objectives set by the European Society of Vascular Surgery which states that reference centers must have, for elective procedures, a mortality rate lower than 2% for EVAR and less than 5% for OR. We also believe that by offering EVAR for these patients with more comorbidities (that would eventually be treated by OR if EVAR had not been introduced), we improved the results of OR itself. We also concluded that age older than 80 years influences the survival curve in the OR group and ASA classification equal or above 4 influences the survival curve in the EVAR group.

Treatment by EVAR has been pointed out as having higher costs in part by the higher rate of re-interventions and our study contradicted this aspect and reinforced the confidence in a costcontainment strategy.

**Table:** 

**Authors' roles & responsibilities**	
RM	Conception and design study; operations and/or trials performance; statistical analysis; analysis and/or data interpretation; manuscript writing or critical review of its content; final manuscript approval
ILA	Analysis and/or data interpretation; manuscript writing or critical review of its content; final manuscript approval
PO	Conception and design study; analysis and/or data interpretation; final manuscript approval
CP	Operations and/or trials performance; analysis and/or data interpretation; manuscript writing or critical review of its content; final manuscript approval
RA	Operations and/or trials performance; analysis and/or data interpretation; manuscript writing or critical review of its content; final manuscript approval
